# A Multimodal Radiation Approach to a Complex Case of Kaposi Sarcoma: Successful Treatment and Long-Term Follow-Up

**DOI:** 10.7759/cureus.92003

**Published:** 2025-09-10

**Authors:** Shyam Jani, Areen Badwal, Nitika Thawani, Shyamal Patel

**Affiliations:** 1 Radiation Oncology, St. Joseph's Hospital and Medical Center, Phoenix, USA; 2 Radiation Oncology, Creighton University School of Medicine, Phoenix, USA

**Keywords:** external beam radiotherapy, hdr brachytherapy, hiv aids, kaposi sarcoma, vmat

## Abstract

Kaposi sarcoma (KS) is an angiogenic tumor frequently seen in HIV-infected patients that involves skin, mucosa, and splanchnic organs. Here, we describe the case of a 57-year-old man with a history of HIV/AIDS on antiretroviral therapy (ART) who presented with KS of the lower extremity. The patient exhibited multiple exophytic lesions circumferentially involving his left leg, causing discomfort and difficulty in activities of daily living. External beam radiation therapy (EBRT) was pursued in lieu of amputation in order to achieve locoregional control while preserving ambulatory function. Due to the extensive longitudinal involvement, the EBRT plan utilized a multi-isocentric volumetric modulated arc therapy (VMAT) technique to cover the entire left leg. The treatment was well tolerated, with a notable response observed in the lesions by the end of the treatment course.

Ten weeks following EBRT, new lesions appeared within the previously treated fields on the left foot. These were treated using high dose-rate (HDR) brachytherapy with a custom skin mold. The patient experienced Grade 3 skin toxicity from treatment, but wound care management led to complete skin regrowth. A second course of HDR brachytherapy was used to treat additional lesions on the mid thigh with no subsequent acute toxicities. Over eight years since initial treatment, the patient has shown no recurrence in the treated areas and remains disease-free. This case illustrates the efficacy and potential long-term benefits of a hybrid multimodal radiation therapy approach in managing KS lesions in patients with HIV/AIDS, particularly when surgical options are limited.

## Introduction

Kaposi sarcoma (KS), an angioproliferative tumor affecting the skin, mucosa, and viscera, requires human herpesvirus 8 (HHV-8) infection for its development [1]. AIDS-related KS is the most prevalent malignancy among individuals living with HIV, with cutaneous lesions commonly manifesting on the lower extremities, face (particularly the nose), oral mucosa, and genitalia [1,2]. These lesions often exhibit an elliptical morphology and may be linearly arranged along skin tension lines in a symmetrical distribution [2].

Although the widespread use of antiretroviral therapy (ART) has led to a global decline in KS incidence, it remains a significant health concern, especially in regions with limited ART access. In the United States, KS incidence among people living with HIV has decreased from approximately 33.3 per 100,000 in 2008 to 4.7 per 100,000 in 2017, though it remains elevated in individuals with CD4 counts below 200 cells/μL (normal range 500-1500), which is a defining criterion for an AIDS diagnosis [3,4].

Treatment strategies for KS prioritize symptom palliation, disease progression prevention, and tumor reduction to mitigate edema, organ dysfunction, and psychological distress [5]. Combination ART is recommended in the management of all patients with AIDS-related KS and may be sufficient as a monotherapy. In some patients, tumor burden may worsen following ART initiation due to immune reconstitution inflammatory syndrome (IRIS), in which immune recovery paradoxically exacerbates inflammatory responses, including KS progression [6]. Chemotherapy, when clinically indicated, can be safely integrated with ART. Local therapies, including surgical resection and radiotherapy (RT), are valuable for cosmetic improvement or managing limited, symptomatic, bulky lesions; however, they do not prevent new lesion formation in untreated areas. The primary role of RT is managing symptomatic disease, either as initial treatment or when the extent of disease precludes surgical resection. Given the radiosensitivity of KS, RT offers significant potential for symptom relief (e.g., pain, bleeding, edema) and local control of cutaneous and mucosal lesions [5].

RT can be delivered using a variety of modalities. External beam radiotherapy (EBRT) involves radiation delivery to a target from a linear accelerator. An advanced EBRT technique is volumetric modulated arc therapy (VMAT), which uses sophisticated computer planning to deliver highly conformal doses to the tumor while minimizing exposure to healthy tissues. High dose-rate (HDR) brachytherapy is another RT modality that involves the temporary placement of a highly radioactive source directly into or adjacent to the tumor, allowing for targeted delivery of radiation.

In the modern ART era, RT continues to play an important adjunctive role in cases with symptomatic, cosmetically disfiguring, or function-limiting KS, particularly when systemic therapy alone proves insufficient [5]. Here, we describe a multi-modal RT approach for a complex presentation of KS on a lower extremity utilizing both EBRT VMAT and HDR brachytherapy, a novel approach that, to the best of our knowledge, has not been described in the literature to date. 

## Case presentation

A 57-year-old male patient with a history of HIV/AIDS presented with a several-month history of progressively worsening pain and drainage from exophytic lesions on his left lower extremity. These lesions, initially noted as nodules on the lower leg, had progressed proximally to involve the thigh. A biopsy performed one month prior at an outside facility confirmed the diagnosis of KS.

**Figure 1:**
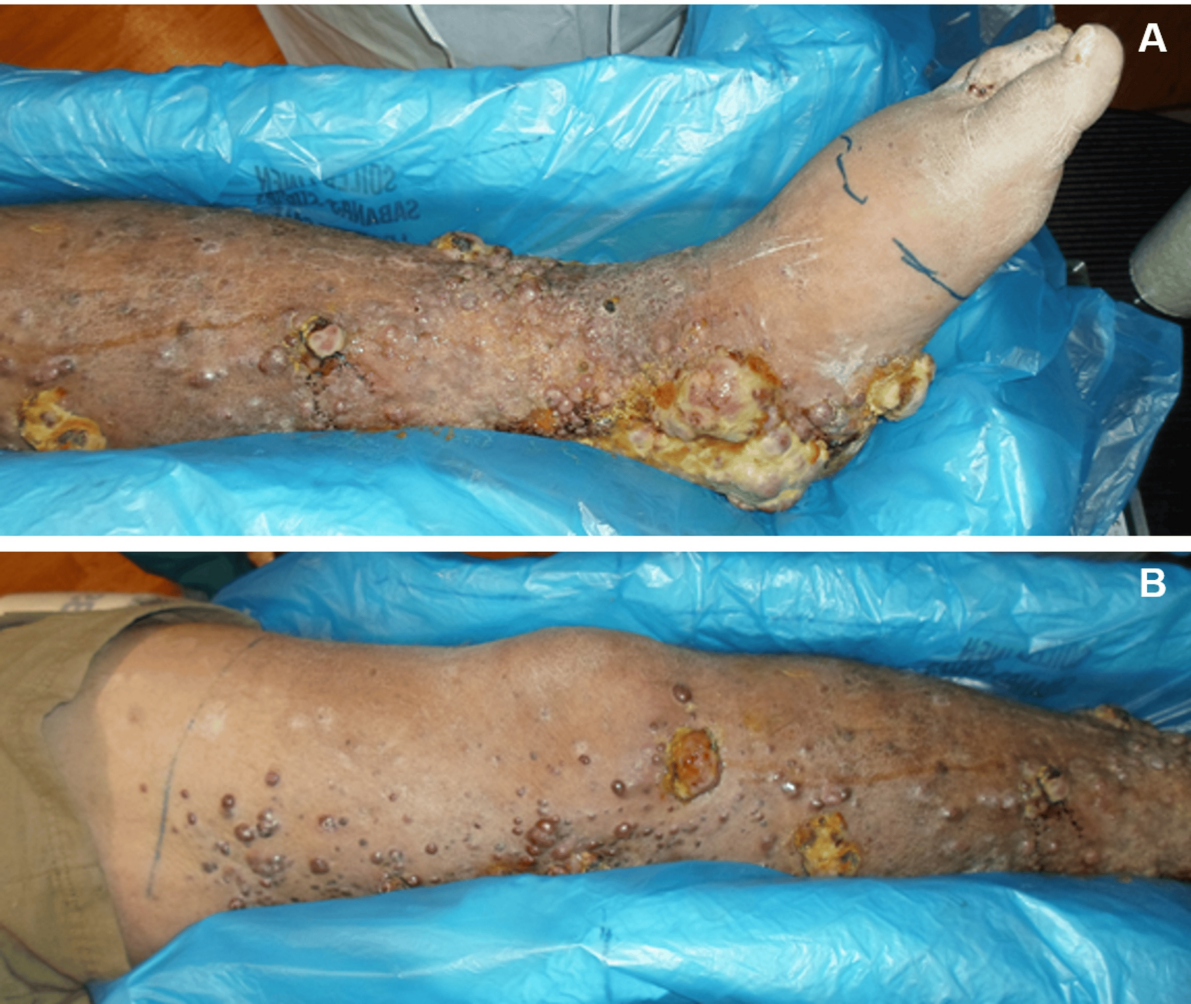
Diffuse circumferential Kaposi sarcoma lesions involving the lower-mid (A) and mid-upper (B) left leg.

The patient initially presented with an extensive number of ulcerative, nodular lesions encompassing his left leg with copious purulent drainage (Figure 1). There was no involvement elsewhere on his body. He reported significant pain, impaired ambulation, and difficulty with hygiene. His CD4 count was 45 cells/µL, and his viral load was 30 copies/mL. He had a history of HIV/AIDS diagnosed in his 30s with a reported history of intermittent ART non-adherence, although he claimed adherence for the preceding three to four months. The patient was afebrile without any overt signs of systemic involvement; empiric antibiotic therapy was initiated to address any potential secondary lower extremity infection. HHV-8 positivity was confirmed with pathology.

Given the extensive disease burden, consultations were obtained from orthopedic surgery, plastic surgery, oncology, and radiation oncology. Debridement and potential amputation were considered by the surgical teams. While the circumferential involvement around the ankle and extensive leg lesions presented significant technical challenges for radiation delivery, RT was recommended due to the radiosensitivity of KS and the limited alternative local treatment options. The patient denied prior RT.

The patient received EBRT with a prescribed dose of 20 Gy in five fractions in 2017. The patient was simulated on a Brilliance Big Bore CT scanner (Philips Inc., Amsterdam, Netherlands) in a feet-first supine position, utilizing a lower body Vac-Lok (CIVCO Radiotherapy, Orange City, IA) with an overlaid 1 cm Superflab bolus (RPD Inc., Albertville, MN) and a Timo headrest (CIVCO Radiotherapy, Orange City, IA) to elevate and stabilize the left ankle. The left leg was centered on the table, and wires were placed on the skin surface by the radiation oncologist to outline the treatment areas. The Vac-Lok and bolus were shaped to the posterior and lateral sides of the left leg, and uncooked rice grains were added to fill in air gaps. A level layer of rice was additionally placed on the anterior leg surface. A planning CT scan was acquired using 3 mm slice thickness with a lateral field-of-view encompassing both legs and the Vac-Lok, and a scan length covering the entire left leg.

**Figure 2:**
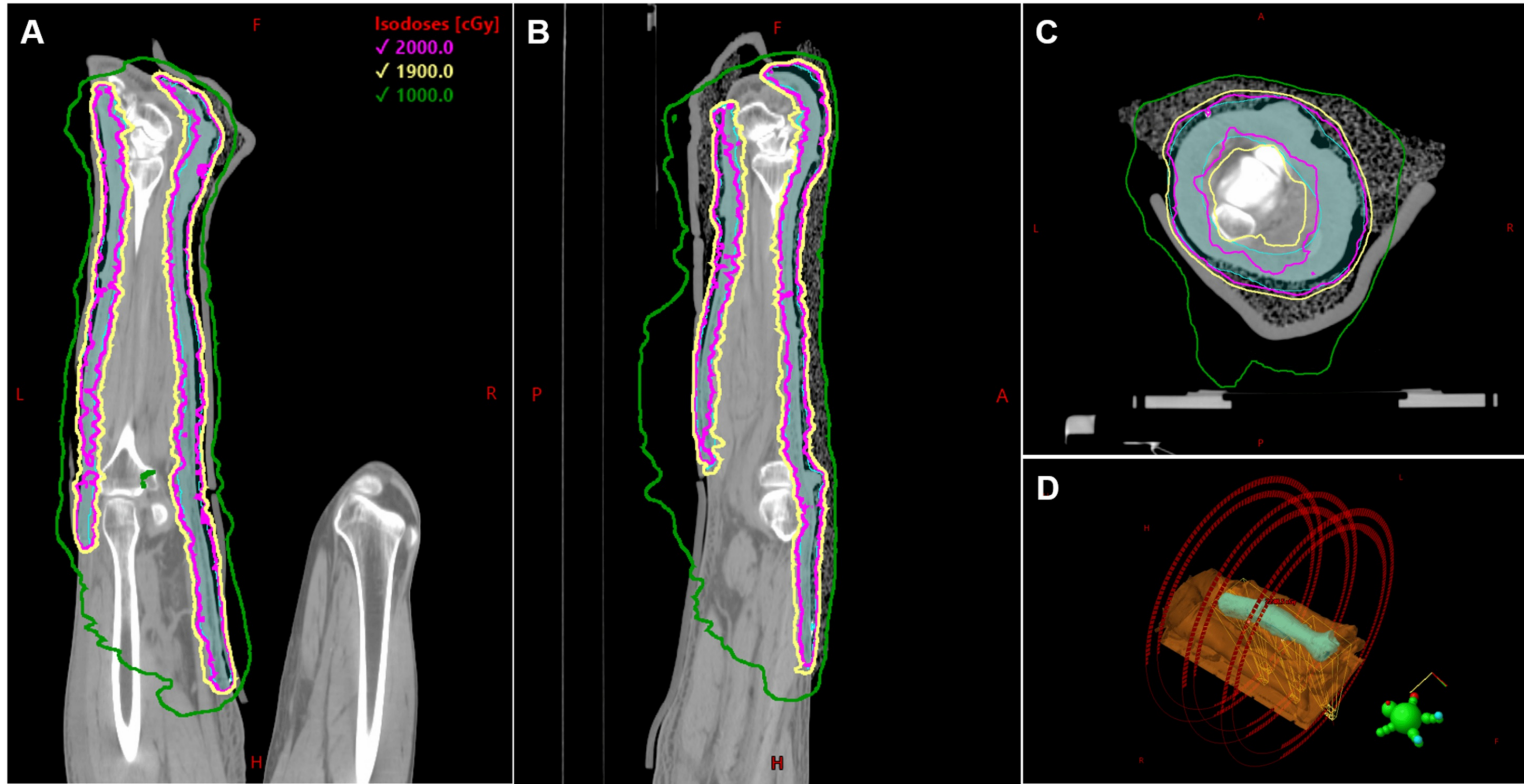
Isodose distribution in the coronal (A), sagittal (B), and axial (C) planes of the CT planning dataset with a prescription (Rx) of 20 Gy in five fractions. The PTV is shown in cyan, and isodose lines of 2000 cGy (Rx dose), 1900 cGy (90% of Rx), and 1000 cGy (50% of Rx) are shown in pink, yellow, and green, respectively. (D) shows a 3D rendering of the body and Vac-Lok (orange) and PTV with the treatment arcs (red curves) split into three isocenters across the superior-inferior extent of the PTV. Avoidance sectors were used to avoid beam entry through the right leg. cGy: centigray; L: left; R: right; A: anterior; P: posterior; H: head; F: foot; PTV: planned target volume

The clinical target volume (CTV) extent was drawn using the wires as a guide and was uniformly expanded by 5 mm to generate the final planning target volume (PTV). A treatment plan was generated using the Eclipse treatment planning system (v11; Varian Medical Systems, Palo Alto, CA) and the AcurosXB v11 calculation algorithm (Varian Medical Systems) using a VMAT technique. Due to the extensive superior-inferior PTV length (>78 cm), three isocenters were placed in the inferior, central, and superior portions of the PTV. Two full arcs with avoidance sectors on the right leg were used for each isocenter, with fields overlapping on the inferior-central and central-superior regions to optimize junction dose homogeneity. All isocenters were optimized in a single plan and subsequently separated into three individual plans for treatment delivery (Figure 2); 95% prescription dose coverage to the PTV was achieved in the final composite plan.

Pre-treatment patient setup involved replicating the CT simulation positioning with the same amounts of rice to fill the lateral/posterior gaps and the anterior surface. Cone beam CTs (CBCTs) were initially acquired at each isocenter for initial leg positioning verification, with repeat CBCTs acquired prior to treatment delivery to ensure accurate positioning. The patient was treated daily over the course of a week. 

The patient tolerated treatment well, showing a clinical response in his lesions by treatment completion. At the two-week follow-up, he reported pruritus and desquamation with multiple lesion detachments. Lesion size, pain, and drainage improved over the subsequent eight weeks, resulting in increased mobility.

**Figure 3:**
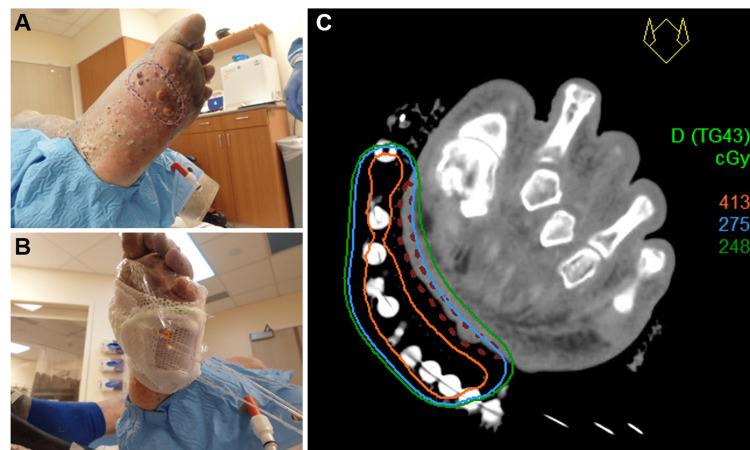
(A) Marker wire surrounding the left sole lesions; (B) Aquaplast mold applicator; (C) a single fractional isodose line distribution on an axial view of the CT planning dataset for the 27.5 Gy in 10 fractions prescription (Rx) for the drawn CTV (dark red dashed lines). Isodose lines for fractional doses of 275 cGy (Rx), 248 cGy (90% of Rx), and 413 cGy (150% of Rx) are shown in blue, green, and orange, respectively. cGy: centigray; CTV: clinical target volume

Ten weeks following completion of EBRT, the patient presented with the development of new, initially asymptomatic lesions. These lesions were located superior to the previously treated area on the left leg, with two additional lesions noted on the sole of the left foot within the irradiated area. After three weeks of observation, the sole lesions became painful and exudative, significantly impacting the patient’s ambulation. Given the clinical progression, a course of HDR brachytherapy with a dose of 27.5 Gy in 10 fractions was prescribed. During CT simulation, the left foot was immobilized using an alpha cradle mold, and a wire was placed around the target lesions. A custom Aquaplast mold (RPD Inc., Albertville, MN) was created with a Freiburg flap applicator using seven catheters, and a CT simulation scan was acquired using a 1.5 mm slice thickness. The CTV was defined using the wire placement as a peripheral guide, and a treatment plan was developed using Oncentra (Elekta, Stockholm, Sweden) with 95% of the prescribed dose covering over 99% of the CTV. The plan was delivered using a Flexitron remote afterloader unit (Elekta) (Figure 3) over two weeks with daily treatment sessions.

After treatment completion, the patient developed a large, painful bulla at the treatment site and moist desquamation, consistent with Common Terminology Criteria for Adverse Events (CTCAE) Grade 3 skin toxicity. Wound care intervention included bulla excision and debridement, resulting in complete re-epithelialization and healing.

Three weeks later, the patient developed new painless lesions on the left upper thigh, two within the previously irradiated EBRT field and four superior to and outside the prior irradiation. A second course of HDR brachytherapy was prescribed, treating the two previously irradiated lesions with 2.75 Gy x 10 fractions and the four unirradiated lesions with 4 Gy x 5 fractions. The setup, simulation, planning, and delivery were similar to those of the left sole treatment. 

**Figure 4:**
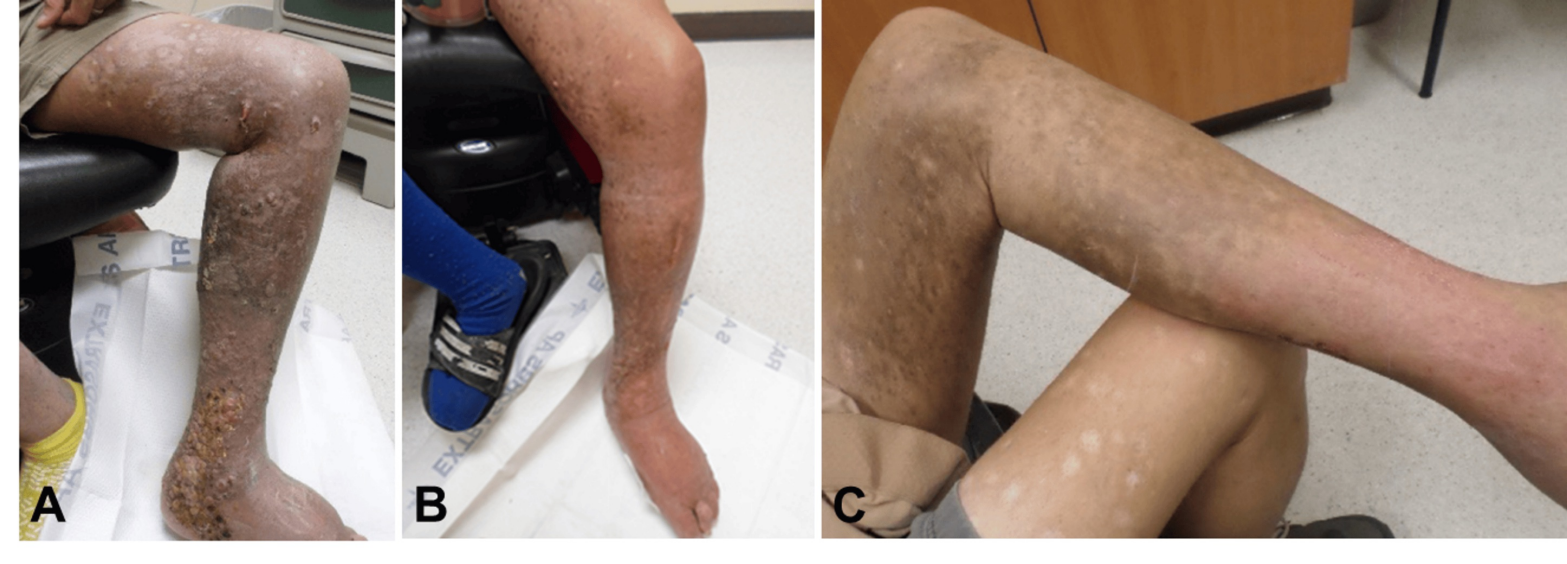
Presentation of Kaposi sarcoma on the left leg (A) two months post EBRT, (B) six months post EBRT, and (C) 19 months post-EBRT and 11 months post HDR. ERBT: external beam radiation therapy; HDR: high dose-rate

Since completion of the second HDR treatment course, the patient has been routinely followed at three- to six-month intervals (Figure 4). The patient developed wound breakdown over his sole one year after HDR, requiring wound care evaluation and management with eventual resolution six months later. He subsequently developed lymphedema involving the left leg that required physical therapy. Nonetheless, eight years post treatment, the patient has continued to remain disease-free with no recurrence within the irradiated areas. 

**Figure 5:**
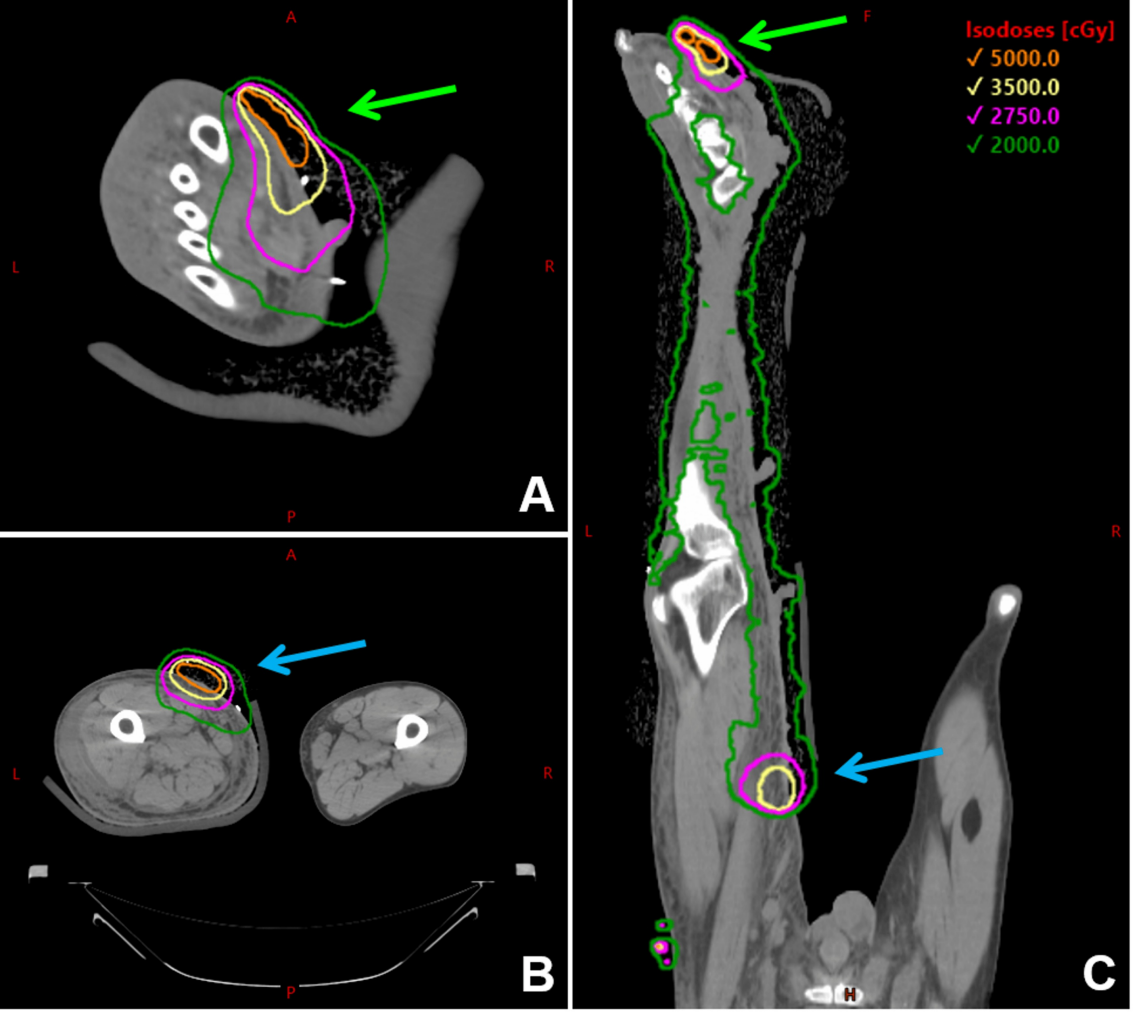
Composite dosimetric overlay with two axial views (A and B) and a coronal view (C) of all treatment courses. In-field recurrences at the left foot and mid-thigh are denoted by light green and blue arrows, respectively. Isodose lines of 5000 cGy, 3500 cGy, 2750 cGy, and 2000 cGy are shown in orange, yellow, pink, and dark green, respectively. cGy: centigray; L: left; R: right; A: anterior; P: posterior; H: head; F: foot

A summary of all treatment courses can be seen in Table 1, and a composite dosimetric overlay of all courses is shown in Figure 5.

**Table 1:** Summary table describing each radiotherapy treatment, treatment description, toxicity, and response EBRT: external beam radiation therapy; VMAT: volumetric modulated arc therapy; HDR: high dose rate; Gy: Gray; CTCAE: Common Terminology Criteria for Adverse Events

Treatment Phase	Year	Modality	Lesion Location(s)	Dose/Fractions	Technique/Setup	Toxicity (CTCAE Grade)	Clinical Response	Time to Response
Course 1	January 2017	EBRT (VMAT)	Left sole to mid-thigh	20 Gy/5	Three isocenters with overlapping arcs delivered concurrently	Grade 2 (pruritus, desquamation)	Mixed partial response (lesion detachment, pain reduction)	Two to eight weeks
Course 2	June 2017	HDR brachytherapy	Sole of the left foot	27.5 Gy/10	Freiburg flap and custom mold	Grade 3 (painful bulla, moist desquamation)	Mixed partial response (wound healing post-debridement)	Two to four weeks
Course 3	September 2017	HDR brachytherapy	Left mid thigh, left upper thigh	27.5 Gy/10 (left mid thigh), 4 Gy/5 (left upper thigh)	Freiburg flap and custom mold	Grade 2 (erythema, dry peeling, hyperpigmentation)	Complete response	Two-four weeks

## Discussion

This case report details the complex management of a 57-year-old male patient with extensive, symptomatic AIDS-related KS, highlighting both the effectiveness and challenges of RT as a monotherapy in this setting. The patient presented with debilitating lower extremity lesions and was offered an amputation. Radiation therapy was recommended instead as a limb-sparing, effective alternative. Despite the technical challenges of multi-isocentric VMAT delivery due to the extensive target volume length, the initial EBRT course resulted in a significant clinical response characterized by pain reduction, decreased drainage, and improved ambulation. We found the Vac-Lok-based setup, using a 1 cm Superflab bolus supplemented with rice grains for additional dose buildup, to be reproducible for each fraction based on pre-treatment CBCT imaging.

Despite the initial clinical response, the patient experienced recurrence both within and outside the irradiated areas. There are published cases of KS reirradiation using different EBRT modalities (photons and electrons) and dose regimens. Tsao et al. [7] had a subset of four reirradiation patients with a 50% partial response at an unspecified follow-up time. Kandaz et al. [8] had a subset of 19 reirradiation patients; at a six-month follow-up, 53% showed complete response while 47% showed stable disease. In a study by Park et al. [9], two patients were re-treated for in-field recurrence; one patient had a complete response for 66 months, while the other patient had a partial response for seven months. Chen et al. [10] showed re-irradiation using VMAT for a single patient with a complete response at a two-month follow-up.

HDR brachytherapy has also been utilized as a single treatment option for localized KS, resulting in high control rates with minimal toxicity [11-12]. To date, a combination of a large field, multi-isocentric EBRT delivery, followed by HDR brachytherapy re-irradiation, has not been reported. The use of customized Freiburg flap applicators with three to four underlying layers of Aquaplast allowed for a suitable separation between the HDR source and lesions, creating a dosimetric falloff that provided adequate target coverage while avoiding excessive surface hotspots. HDR was effective in managing localized disease progression, although subsequent skin toxicities and wound care interventions highlight the importance of careful monitoring and management post-treatment. 

There remain no clear and comprehensive guidelines for the optimal radiation management of KS. A prior study found 80% to 100% symptomatic relief across various types of KS and external beam regimens when utilizing doses as low as a single fraction of 8 Gy up to doses as high as 30 Gy in non-AIDS-related patients [13]. Stelzer et al. found fractionated regimens using doses of 20-40 Gy to yield superior response rates (80%) than a single fraction 8 Gy regimen (50%) in AIDS-related patients [14]. At our institution, 20 Gy in five fractions (resulting in an equivalent dose in 2 Gy fractions (EQD2) of 23 Gy and a biologically effective dose (BED) of 28 Gy, using an alpha beta ratio of 10) is the recommended treatment course when feasible after accounting for lesion size and location along with patient functional status and logistics, as comparable control rates have been found with this fractionation compared to higher doses [7, 13]. Lower doses per fraction in a longer course may be utilized in treating reirradiation cases in locations with less perfusion, such as the lower extremities, to improve normal tissue sparing. In this case, a prescription of 27.5 Gy in 10 fractions (EQD2 of 29 Gy, BED of 35 Gy) was utilized for the HDR brachytherapy treatment of recurrent disease. 

When considering radiation therapy for KS, a variety of modalities are available for treatment. As discussed earlier, electrons or HDR brachytherapy with a custom skin mold can be utilized for superficial lesions, while photons are used for the treatment of deeper or more extensive lesions. Similar to the approach in sarcoma patients, we aim to spare a strip of skin when treating larger or more extensive lesions in extremities to allow for lymphatic drainage preservation. The strip should be 1 to 2 cm in length, and we utilize the constraint V20 Gy < 50% per Radiation Therapy Oncology Group (RTOG) 0630 [15]. As long as the skin surface is conducive to a conformal mold formation, we favor HDR brachytherapy for superficial lesions <5-10 cm in size or in recurrent disease, as it allows for the least dose to surrounding normal tissue. We utilize EBRT for deeper lesions or in situations of extensive disease, such as in the case of this patient’s initial presentation.

Long-term follow-up is needed in managing KS patients, particularly those with large or recurrent lesions. Identifying and addressing skin breakdown, lymphedema, and other potential toxicities early on can lead to improved functional outcomes.

## Conclusions

The use of multimodal radiation therapy incorporating multi-isocentric EBRT with VMAT followed by HDR brachytherapy reirradiation may offer a feasible and effective option in selected cases of KS presenting with HIV/AIDS. While 20 Gy in five fractions (EQD2 of 23 Gy) was utilized for the initial course of untreated lesions and led to significant tumor response, an additional dose of 27.5 Gy in 10 fractions (EQD2 of 29 Gy) was successfully utilized for lesions that recurred within the treatment field. This led to overall disease control for eight years. In this patient, the combination of VMAT-based EBRT followed by focal HDR brachytherapy achieved tumor control and symptom palliation and avoided limb amputation, highlighting a potential treatment pathway in anatomically complex, radiosensitive cases of KS. Further studies would help to optimize the use of RT in KS, especially in patients with extensive disease involvement. 
